# The association between socio-demographic characteristics and adherence to breast and colorectal cancer screening: Analysis of large sub populations

**DOI:** 10.1186/1471-2407-11-376

**Published:** 2011-08-25

**Authors:** Rachel Wilf-Miron, Ronit Peled, Einat Yaari, Anna Vainer, Avi Porath, Ehud Kokia

**Affiliations:** 1 Quality Management in Health Care, Maccabi Healthcare Services, Tel-Aviv, Israel; 2 Faculty of Management, Tel-Aviv University, Tel-Aviv, Israel; 3 Faculty of Health Sciences, Ben Gurion University of the Negev, Beer Sheva, Israel; 4 Central Management, Maccabi Healthcare Services, Tel-Aviv, Israel

**Keywords:** Breast Cancer, Colon Cancer, Early Detection of Cancer, Health Disparities, Demographic Characteristics

## Abstract

**Background:**

Populations having lower socioeconomic status, as well as ethnic minorities, have demonstrated lower utilization of preventive screening, including tests for early detection of breast and colorectal cancer.

**The objective:**

To explore socio-demographic disparities in adherence to screening recommendations for early detection of cancer.

**Methods:**

The study was conducted by Maccabi Healthcare Services, an Israeli HMO (health plan) providing healthcare services to 1.9 million members. Utilization of breast cancer (BC) and colorectal cancer (CC) screening were analyzed by socio-economic ranks (SERs), ethnicity (Arab vs non-Arab), immigration status and ownership of voluntarily supplemental health insurance (VSHI).

**Results:**

Data on 157,928 and 303,330 adults, eligible for BC and CC screening, respectively, were analyzed. Those having lower SER, Arabs, immigrants from Former Soviet Union countries and non-owners of VSHI performed fewer cancer screening examinations compared with those having higher SER, non-Arabs, veterans and owners of VSHI (p < 0.001). Logistic regression model for BC Screening revealed a positive association with age and ownership of VSHI and a negative association with being an Arab and having a lower SER. The model for CC screening revealed a positive association with age and ownership of VSHI and a negative association with being an Arab, having a lower SER and being an immigrant. The model estimated for BC and CC screening among females revealed a positive association with age and ownership of VSHI and a negative association with being an Arab, having a lower SER and being an immigrant.

**Conclusion:**

Patients from low socio-economic backgrounds, Arabs, immigrants and those who do not own supplemental insurance do fewer tests for early detection of cancer. These sub-populations should be considered priority populations for targeted intervention programs and improved resource allocation.

## Background

Social justice affects the way people live and, in consequence, their chances of illness and risk of premature death. Avoidable health disparities result from the circumstances in which people grow up, live, work and age, as well as what systems put in place to deal with health and disease [1].

During the last decade, disparities in health care have entered the emerging debate over quality of care. In 2001, the Institute of Medicine (IOM) declared that among other dimensions, quality of health care is also defined by it being equitable, i.e., care that does not vary in quality because of personal characteristics such as gender, ethnicity, geographic location and socioeconomic status [2]. This designation of equality as a dimension in quality of care reinforced the commitment of health organizations to reduce health disparities among all populations. A report published by the IOM in 2002 suggested that awareness of the health care gap be heightened among sectors that include health care providers, their patients and society at large [3].

Disparities in health outcomes and healthcare access and utilization, based on ethnicity and socio-economic status, are well documented throughout the world [3-6] as well as in Israel [7-10]. These disparities exist despite the fact that Israel has a universal health care system, guaranteed under the National Health Insurance Law (NHIL), formally declared to be based on the principles of equality, justice and social solidarity. The system is financed through taxation linked to income and general revenue. Health care is provided by four non-profit health plans. Each plan is required to provide its members with a basic benefits package that includes community-based care as well as hospitalization. Choice of health plans is autonomous, with prohibition against denial of enrollment.

Preventive care, such as screening for early detection of breast and colorectal cancer, is included in a comprehensive benefit package of services and are free of charge in all the health plans. In addition, all Israeli HMOs offer their beneficiaries the opportunity to purchase, on a voluntarily basis, supplementary health insurance to expand the basket of benefits. The tariff is age-based and not related to health status.

Maccabi Healthcare Services (MHS) is an Israeli health plan providing community-based health services throughout the country to 1.9 million members from diverse population groups. Services are provided in five geographical regions through 150 local branches (the basic administrative unit), and is based on a core staff of some 4,000 independent physicians and 1,000 nurses. Physicians are self-employed, working from either MHS clinics located in the branches or their private offices, usually in solo practices. Nurses, other health professionals and administrative staff are salaried and generally work in the branches.

Since 2004, MHS has been measuring performance in 6 clinical domains. Among the 24 quality measures used are screening tests for the early detection of two malignant diseases: Breast Cancer (BC) and Colorectal Cancer (CC). Results of performance measures are analyzed and reported internally on a monthly basis to central headquarters, regions and branches. Reporting transparency has brought to light disparities between regions and branches that serve different populations and raised recognition of inequity as a significant issue, to the point where it gradually entered the organizational discourse. Field staff have since identified local barriers to quality care and constructed tailor-made interventions that have effectively reduced disparities, such as the case of mammography among Arab women [11].

*Mammography screening for early detection of BC *has been shown to effectively reduce mortality in women aged 50-74 regardless of screening interval or number of mammographic views per screen [12,13]. Screening has thus become a major recommended practice in health organizations throughout the world as well as in Israel [14]. Disparities in mammography use among Israeli sub populations was previously documented in two studies, the first a cross-sectional study initiated by MHS [15], the second conducted by the largest Israeli HMO (Clalit Health Services) [16]. In the first study, Baron Epel and her colleagues suggested that the interventions implemented by MHS may have increased mammography utilization but had not completely eliminated disparities between member sub populations. The second study indicated the same pattern. These findings can be confirmed with Israeli CDC data, which show large differences in rates of self-reported mammography use by our sub population groups [17].

*Screening for early detection of CC*: Such screening is also universally recommended. Annual fecal occult blood testing (FOBT) and periodic colonoscopy (every 5 years) are the common methods for CC screening among normal-risk populations [18]. In Israel, FOBT has been promoted nationally as the recommended primary method for screening. Since an increasing number of health consumers and caregivers prefer colonoscopy as their screening method of choice, both methods are included in the measure [14]. Despite recent increases in colorectal screening due to national programs as well as initiatives to promote this preventive care introduced by all four HMOs, disparities in early detection of colon cancer are still documented [14,16].

In 2008, MHS took a strategic decision to invest in a long-term effort to reduce disparities in health care and health outcomes. The first step was to float core data about differences in performance measures among MHS members belonging to different population groups [19]. To this end, a methodology and designated database for monitoring and ongoing analysis was constructed [20].

The hypothesis tested in the research stated that despite the fact that breast and colorectal cancer screening in Israel is free of charge, other social and cultural barriers influence utilization of these important examinations. Hence, the current research objective was to analyze the association of socio-economic status, immigration, ethnicity (Arab vs. non-Arab) and VSHI ownership with utilization of cancer screening tests.

## Methods

### Setting

The study was conducted by MHS.

### Study Period

Data was extracted in November 15, 2008 (Due Day).

### Study Population

All MHS adult members who visited their general practitioner (GP) at least once during the previous two years were eligible for inclusion in the data set. We used the latter criterion as a proxy for a routine patient-physician relationship in all measures that reflect care at the primary-care level. Previous analyses had revealed that 6% of MHS members, aged 50-74, who had not visited their GP in a 2-year period, demonstrate far less-favorable health outcomes when compared with those who have regular interactions with their physician; this sub-population should therefore be analyzed separately.

### Eligibility

BC screening: Women aged 52-74, (52 is the age when a woman is expected to have utilized mammography screening during the previous two years, i.e., since reaching the age of 50); CC screening: men and women aged 51-74. MHS members who had been diagnosed with breast or colorectal cancer in the past were excluded from the analysis.

### Data sources

Data were extracted from: (1) the MHS computerized billing system; (2) the MHS computerized Performance Measurement System; (3) the Israeli Census for data on socio-economic status ranks (SER) and ethnicity (Arab vs non-Arab [21].

## Definitions

### Performance measures

#### BC Screening

Eligible women who had undergone a mammography test at least once during the two previous years.

#### CC Screening

Eligible men and women who had transmitted a sample for FOBT at least once during the previous year or had undergone at least one colonoscopy during the previous five years.

### Independent Variables

#### Socio Economic Rank (SER)

Israel is administratively divided into sub-districts, each having a population of at least 3,000 residents; each sub-district tends to be relatively homogeneous. Sub-districts are characterized by their socio-economic status, based on variables such as housing density, employment, income, education, and so forth. These ranks are updated every 15 years when a national population survey is conducted. We used the latest information with which sub-districts are ranked on a 1-20 scale (1-lowest, 20-highest) obtained from the 1995 national population survey and census, carried by the Central Bureau of Statistics [21]. As part of our analysis, subjects were allocated to a sub-district according to his/her last recorded address and then assigned a socio-economic status, which was later collapsed into four SES rank (SER) categories: 1-5, 6-10, 11-15, and 16-20. It is important to note that the SER given to a MHS member was based on residential area or sub-district rather than personal information.

#### Ethnicity (Arab vs. Non-Arab)

Israeli Arabs comprise 20% of overall population [21]. They live in rural and urban, Arab and mixed localities throughout the country. All Israeli Arabs are citizens and entitled to equal health care services under the NHIL. Personal data on ethnicity or income is not transmitted to HMOs in order to avoid discrimination or adverse selection of members. Therefore, Israeli Arabs can be identified only by their residential locality or (as in large mixed cities such as Nazareth) sub-district data, recorded by the National Census [21]. Israeli research uses this data in cases where the direct questioning of subjects is not possible [16,22]

#### Immigration

Starting in 1990, Israel became home to a wave of immigration (about 1 million people) from countries belonging to the Former Soviet Union (FSU), who contributed to a 15% growth in total population during that decade. The MHS data set contains immigration status for FSU immigrants only. Hence, we included only these immigrants under the category "Immigration". We should note that total MHS members belonging to the other immigrant groups arriving in recent decades are too few in number for inclusion in this analysis as a distinct group.

#### VSHI

Although all Israeli citizens are insured for health care, Israeli HMOs offer their members the opportunity to purchase voluntary supplemental health insurance (VSHI), meant to enlarge the benefits package. Every member, irrespective of health status, can purchase VSHI; the tariff is age-based and unrelated to health status [23]. According to reports published by the Israeli Ministry of Health, 74.4% of all Israelis and 87.8% of MHS beneficiaries own VSHI [24]. MHS members who own this type of insurance tend to have a higher SER. The lack of VSHI may therefore be regarded as a proxy for low SER.

#### Data set construction

We combined MHS Billing System, PMS and Israeli Census data into one data set using the AS400 query system.

#### Statistical analysis

For all eligible subjects, we performed (A) an analysis of variance for BC and CC screening between sub-groups (gender, SERs, ethnicity, immigration and VSHI) using Chi-square techniques; (B) Logistic Regression Models were estimated for three dependent variables: (1) BC screening (2) CC screening and (3) BC and CC screening only for females. The independent variables were included in the models only if they reached the required significance level (p<0.05) in the univariant analysis. Although we had compared four ranks in the univariant analysis, we combined SER 1-10 and 11-20 into two separate dummy variables in the final models. Our decision was based on the preliminary models indicating robust results only for the two more-inclusive (1-10 and 11-20) rather than the four less-inclusive (1-5; 6-10; 11-15; 16-20) ranks. Odds ratios (OR) and 95% confidence intervals (95%CI) were also calculated.

### Ethics

The data used for this study are not openly available; hence, the study did not require ethical approval

## Results

We analyzed 157,928 women who were eligible for BC screening and 303,330 men and women who were eligible for CC screening tests. Utilization of mammography screening was more prevalent in the higher SERs (11-15, 16-20): 68.6% and 72.9%, respectively, compared to the lower ranks (1-5, 6-10): 64.1% and 65.6%, respectively (p < 0.001). Mammography tests was less utilized by Arab women compared to non-Arabs: 61% and 69.1%, respectively (p < 0.001); by veteran residents compared to immigrants: 69.3% and 67.1%, respectively (p < 0.001); and by owners of VSHI compared to non-owners: 70.9% and 51.5%, respectively (p < 0.001) (Table 1).

**Table 1:** Utilization (%) of Breast and Colon Cancer Screening by Gender, SER, Immigration, Ethnicity and VSHI

*Variable*	*BC^1 ^Screening*	*P Value*	*CC^2 ^Screening*	*P Value*
Eligible (N)	157,928		303,330	
Age	52-74		51-74	
***Gender***				
Female	68.8	---	29.0	< 0.411
Male	---	---	28.9	
***SER***				
1-5	64.1		21.7	
6-10	65.6	< 0.001	25.0	< 0.001
11-15	68.6		28.8	
16-20	72.9		34.4	
***Ethnicity***				
Arabs	61.0	< 0.001	20.4	< 0.001
Non Arabs	69.1		29.3	
***Immigration***				
Immigrants	67.1	< 0.001	25.1	< 0.001
Veterans	69.3		30.0	
***VSHI***				
Owners	70.9	< 0.001	30.3	< 0.001
Non Owners	51.5		18.1	

Notes: ^1 ^Breast Cancer; ^2 ^Colon Cancer

The final logistic regression model for BC screening as a dependent variable revealed a weak positive association with age (OR: 1.004; 95%CI: 1.002-1.005) and a robust positive association with being an owner of VSHI (OR: 2.235; 95%CI: 2.155-2.319). Negative associations were found with being an Arab women (OR: 0.883; 95%CI: 0.813-0.959) and belonging to a low SER (1-10) (OR: 0.883; 95%CI: 0.860-0.907). No significant association was found with being an immigrant from the FSU and BC screening (Table 2).

**Table 2:** Results of Final Regression Model for Dependent Variable: Utilization of Mammography for Breast Cancer Screening (Women only)

*Variable*	*B*	*SE^1^*	*p Value*	*OR^2^*	*95% CI^3^*
***Age***	0.994	0.001	< 0.001	1.004	1.002-1.005
***Ethnicity (Arabs)***	-0.124	0.042	0.003	0.883	0.813-0.959
***SER 1-10***	-0.125	0.014	< 0.001	0.883	0.860-0.907
***Immigration***	0.019	0.015	0.188	1.02	0.991-1.049
***VSHI***	0.804	0.019	< 0.000	2.235	2.155-2.319

Notes: ^1 ^Standard Error; ^2 ^Odds Ratio; ^3 ^95% Confidence Interval

The final logistic regression model for CC screening as a dependent variable revealed a positive association with age (OR: 1.038; 95%CI: 1.036-1.039) and a positive and robust association with owning VSHI (OR: 1.824; 95%CI: 1.757-1.885). Negative associations were found with being an Arab (OR: 0.789; 95%CI: 0.735-0.846), belonging to a lower SER (1-10) (OR: 0.774; 95%CI: 0.758-0.791) and with being an immigrant from the FSU (OR: 0.832; 95%CI: 0.814-0.851). No statistical association was found with gender (Table 3). We estimated a third model for eligible females where the dependent variable was utilizing BC and CC screening tests. This model revealed a weak positive association with age (OR: 1.036; 95%CI: 1.034-1.038) and a robust positive association with owning VSHI (OR: 1.991; 95%CI: 1.891-2.096). A negative association was found with being an Arab women (OR: 0.887; 95%CI: 0.798-0.986), having lower SER (1-10) (OR: 0.785; 95%CI: 0.762-0.810) and being an immigrant from the FSU (OR: 0.902; 95%CI: 0.873-0.932) (Table 4).

**Table 3:** Results of the Final Regression Model for Dependent Variable: Utilization of Colorectal Cancer Screening (Men and Women)

*Variable*	*B*	*SE^1^*	*p Value*	*OR^2^*	*95% CI^3^*
***Age***	0.037	0.001	< 0.001	1.038	1.036-1.039
***Gender (male)***	-0.012	0.009	< 0.187	1.012	0.994-1.030
***Ethnicity (Arabs)***	-0.237	0.036	< 0.001	0.789	0.735-0.846
***SER 1-10***	-0.256	0.011	< 0.001	0.774	0.758-0.791
***Immigration***	-0.183	0.012	< 0.001	0.832	0.814-0.851
***VSHI***	0.601	0.017	< 0.001	1.824	1.756-1.885

Notes: ^1 ^Standard Error; ^2 ^Odds Ratio; ^3 ^95% Confidence Interval

**Table 4:** Results of Final Regression Model for Dependent Variable: Performing CC Screening and BC Screening (Women).

*Variable*	*B*	*SE^1^*	*p Value*	*OR^2^*	*95% CI^3^*
***Age***	0.036	0.001	< 0.001	1.036	1.034-1.038
***Ethnicity (Arabs)***	-0.120	0.054	0.026	0.887	0.798-0.986
***SER 1-10***	-0.241	0.016	< 0.001	0.785	0.762-0.810
***Immigration***	-0.103	0.017	< 0.001	0.902	0.873-0.932
***VSHI***	0.688	0.026	< 0.001	1.991	1.891-2.096

^1 ^Standard Error; ^2 ^Odds Ratio; ^3 ^95% Confidence Interval

## Discussion

Our study identified those population sub-groups who demonstrate less favorable health measures regarding preventive care: men and women who belong to lower socio-economic ranks, Arabs, who are known to be a deprived Israeli ethnic minority, immigrants and people who have not purchased VSHI. These MHS members should be considered as priority populations for intervention programs and resource allocation to increase utilization of screening tests.

These findings are interesting due to the fact that Israel has a universal health system and that the studied examples of preventive care are highly accessible and completely free of charge. In this case, affordability cannot explain the variations in service uptake.

In addition, eligible MHS members are subject to various outreach efforts by our staff (physicians, nurses, other health professionals as well as administrative staff) to promote service utilization, such as mail invitations to perform BC screening, telephone reminders, scheduled appointments for mammography [11] and transmission of FOBT kits at MHS branches when members come for routine laboratory tests. Despite these efforts, disparities still exist.

Our results are compatible with national statistics pertaining to the national program for community quality indicators, which has demonstrated low utilization of BC but not of CC screening among persons having low socioeconomic status [14]. In addition, our results are compatible with those from other countries with universal health systems and national preventive care programs [25-28]. In these studies, it has been argued that innovations in health technology may widen inequalities if people with more knowledge, money or power have a greater ability to harness the beneficial effects [29,30].

In low socio-economic populations, more than affordability is required to take advantage of care availability in general and preventive services in particular. Several barriers, internal to the individual as well as within their physical and social environment, hamper such behavior. Moreover, even if environments are supportive, making positive healthy choices is rather difficult for people do not feel in control over their circumstances [31]

Baron Epel, an Israeli researcher, has suggested in her two recently published studies that subjective norms, fatalism, fear of breast cancer, and perceived effectiveness are associated with mammography use. These associations were not persistent with all the sub-population groups she surveyed (Orthodox and secular Jews, Immigrants, Arabs). Each population seems to exhibit unique values and norms, the presence of which can predict preventive behavior [32,33]. The main conclusion emerging from these studies is that the behavioral factors relevant for each group must be investigated in-depth to better understanding how to most effectively target organizational resources and thus reduce disparities in preventive care. Furthermore, it appears that tailor-made interventions formulated and delivered by local teams who are familiar with the specific community's norms, beliefs and values are likely to be very effective [11].

Lessons learnt from a National Cancer Screening Program in the UK, show that variations in the uptake of breast cancer screening were closely related to social deprivation. These findings support the theory that societies create and shape patterns of health and disease. Health care innovations are generally introduced within the context of inequalities that shape the distribution of the health benefit, thereby affecting morbidity patterns [26,29,34,35].

Both breast and cervical cancer screening require attendance at a clinic; hence, factors such as time pressures, transport problems or discomfort in interacting with medical services and health professionals could also lie behind some of the socioeconomic differences in utilization [36]. In some countries, a "Patient Navigation" (PN) method has been implemented, mainly for deprived populations, to assist people in obtaining cancer information, screening, treatment, and support services. This method provides people from weak segments of the populations with a "bridge" facilitating their interaction with the healthcare system. When combined with additional methods, such as educational programs, overall effectiveness may increase [37].

At this point we can conclude that multifactor interventions that target more than one aspect of the screening process are likely to have greater effects. The largest challenge for future research will be to identify those methods that address the moral, cultural, educational and other personal resources involved in motivating screening uptake.

Our findings suggest that MHS members who did not purchase VSHI were less likely to perform screening tests. Lack of VSHI is an indicator of deprivation, and not a causal factor for lower utilization of care since VSHI ownership does not affect accessibility or affordability of BC and CC screening tests.

Israeli Arabs belong to an ethnic minority representing about 20% of Israel's current population. They generally belong to the lowest socioeconomic ranks. As Israeli citizens, they are entitled to equal health services. However, previous studies indicate that this sub-population suffers from major disparities in health care utilization and health outcomes [11,14]. A study conducted by an Israeli group of researchers in one of northern Israel's largest hospitals providing services to a major proportion of Arabs suggests that Arab patients were more likely to be obese and to have diabetes. A greater percentage of Arab patients also suffered higher rates of stroke [38]. However, tailored-made interventions to increase use of mammography by Arab women [11] reduced but did not completely eliminated differences between Arabs and other Israeli ethnic groups [15].

Recent studies conducted with participants from the Palestinian Authority and from Israel have reported a combination of personal, cultural, normative and conceptual factors that act as barriers to BC screening [39]. The begged-for conclusion is that any intervention aimed at this ethnic sub population must be tailor-made.

With respect to immigration, our study identified immigrants from FSU countries as a population at risk for lower utilization of cancer screening. This group is characterized by its high educational level, relatively good assimilation into Israeli society and relatively long residence (10 years and more) at the time of the study. An earlier study carried out by Remennick and colleagues suggests that although FSU immigrant women acknowledged the personal risks and understood the role of screening, they still avoided taking preventive action [40]. In 2003, the same author demonstrated the low priority of preventive health on the personal agenda of female immigrants from the FSU, who appeared to be burdened by more immediate survival needs (income, housing, care of other family members, etc.). Other barriers identified were the lack of referrals from primary care providers, fear of cancer diagnosis, apprehensions regarding irradiation and the pain associated mammography, a generally fatalistic attitude towards health and illness, and mistrust of current cancer therapy [41]. These studies indicate that personal barriers may also play a major role in cancer screening utilization.

Our study has therefore identified priority populations for special organizational attention and efforts aimed at reducing health disparities. A methodology for the investigation of barriers to equitable care and tailoring solutions to these barriers has been implemented and tested since early 2010. Among the key factors distinguishing this methodology are: (1) Training of MHS staff in cultural competency in order to better understand the social, ethnic, religious needs and preferences of unique populations. In this respect we view poverty as a culture that influences one's health choices and preferences; (2) Translation of educational materials regarding preventive care into Arabic and Russian in order to overcome language barriers. For better linguistic accessibility, an organization-wide medical interpreting and translation service, using low-tech technology (the telephone) has recently been implemented; and (3) Increased accessibility of preventive services, obtained by using methods such as a mobile mammography unit, which is sent to remote and rural areas where transportation difficulties create barriers.

### The study's added value

First, disparities in utilization of preventive care are well-documented. However, in Israel, the current research is one of very few attempts to describe this phenomenon through analysis of an extensive database hosted by a large HMO. This study therefore sheds greater light on disparities existing in a society where financial barriers to preventive care do not exist.

Second, in a world where health organizations are challenged by wise allocation of scarce resources, it is important to carefully identify priority populations. This study helped MHS to redefine its organizational strategy regarding how every decision is made by filtering the process through the lens asking how much the decision will contribute to disparities reduction.

## Limitations

### Eligibility

Our subjects were eligible for participation in the study if they had visited their GP at least once during the previous two years. Six percent of our beneficiaries did not meet this criterion. We do not consider this to be the result of a selection bias since previous studies have suggested that this group demonstrated unique health outcomes characteristics and therefore should be investigated separately.

### Ethnicity

The ethnic identity of members is not available to HMOs for historical reasons, mainly to avoid discrimination. Hence, we identified Arab members by area of residence. We estimate that 90% of this sub population was thereby identified and thus participated in our study. We should note that Israeli Arabs live mainly in almost exclusively Arab settlements. For those who live in mixed cities such as Nazareth, we obtained information about sub-districts, which include populations of 3,000 inhabitants, while assuming that neighborhoods are more or less homogeneous.

### SER

Because data on socio-economic variables such as education, income and housing conditions are not directly available to Israeli HMOs on the personal level, we could classify our members only according to the SERs assigned to their locality. Undoubtedly, averaging the values of these variables for a 3,000-member sub district can never be as accurate as personal datasets. Analysis of the Israeli data supports the validity of using district SER as a proxy for personal SER [42].

## Conclusion

Preventive care has been positively associated with socioeconomic status and the ownership of voluntarily supplemental health insurance, and negatively associated with immigration and Arab ethnicity. These factors do not, by themselves, fully explain low utilization of preventive health care services. Hence, the other factors that tentatively influence healthy behavior and lifestyle should be investigated in depth before planning any intervention program.

## Competing interests

The authors declare that they have no competing interests.

## Authors' contributions

RWM was the principal investigator and participated in each step of the manuscript preparation; RP carried out the statistical analysis and participated in writing the manuscript; EY participated in the design of the study and in the sequence alignments; AP helped draft the manuscript and participated in the sequence alignment; EK helped draft the manuscript and participated in the sequence alignment; All authors read and approved the final manuscript.

## Pre-publication history

The pre-publication history for this paper can be accessed here:

http://www.biomedcentral.com/1471-2407/11/376/prepub
